# Molecular Assessment of *Staphylococcus Aureus* Strains in STAT3 Hyper-IgE Syndrome Patients

**DOI:** 10.1007/s10875-022-01293-7

**Published:** 2022-06-02

**Authors:** Vera Schwierzeck, Renate Effner, Felicitas Abel, Matthias Reiger, Gundula Notheis, Jürgen Held, Valeska Simon, Sebastian Dintner, Reinhard Hoffmann, Beate Hagl, Johannes Huebner, Alexander Mellmann, Ellen D. Renner

**Affiliations:** 1 Translational Immunology in Environmental Medicine, School of Medicine, Technical University of Munich, 81675 Munich, Germany; 2 Institute of Environmental Medicine, Helmholtz Zentrum Munich, 85764 Neuherberg, Germany; 3 Institute of Hygiene, University Hospital Münster, 48149 Münster, Germany; 4 University Children’s Hospital, Dr. von Haunersches Kinderspital, Ludwig Maximilian University, 80337 Munich, Germany; 5 Department of Environmental Medicine, Medical Faculty of University Augsburg, 86156 Augsburg, Germany; 6 Mikrobiologisches Institut - Klinische Mikrobiologie, Immunologie und Hygiene, Universitätsklinikum Erlangen und Friedrich-Alexander-Universität (FAU) Erlangen-Nürnberg, 91054 Erlangen, Germany; 7 Institute for Laboratory Medicine and Microbiology, University Hospital Augsburg, 86156 Augsburg, Germany; 8 Institute of Pathology, University Hospital Augsburg, 86156 Augsburg, Germany; 9 Department of Pediatrics, Klinikum rechts der Isar, School of Medicine, Technical University of Munich, 80804 Munich, Germany

**Keywords:** *Staphylococcus aureus (S. aureus)*, STAT3 hyper-IgE syndromes (STAT3-HIES), whole genome sequencing (WGS), protein A (*spa*) typing, immune evasion cluster (IEC)

## Abstract

Hyper-IgE syndromes (HIES) are a group of inborn errors of immunity (IEI) caused by monogenic defects such as in the gene *STAT3* (STAT3-HIES). Patients suffering from HIES show an increased susceptibility to *Staphylococcus aureus* (*S. aureus*) including skin abscesses and pulmonary infections. To assess if the underlying immune defect of STAT3-HIES patients influences the resistance patterns, pathogenicity factors or strain types of *S. aureus*. We characterized eleven *S. aureus* strains isolated from STAT3-HIES patients (*n* = 4) by whole genome sequencing (WGS) to determine presence of resistance and virulence genes. Additionally, we used multi-locus sequence typing (MLST) and protein A (*spa*) typing to classify these isolates. Bacterial isolates collected from this cohort of STAT3-HIES patients were identified as common *spa* types in Germany. Only one of the isolates was classified as methicillin-resistant *S. aureus* (MRSA). For one STAT3 patient WGS illustrated that infection and colonization occurred with different *S. aureus* isolates rather than one particular clone. The identified *S. aureus* carriage profile on a molecular level suggests that *S. aureus* strain type in STAT3-HIES patients is determined by local epidemiology rather than the underlying immune defect highlighting the importance of microbiological assessment prior to antibiotic treatment.

## Introduction

While *Staphylococcus aureus* (*S. aureus*) frequently colonizes skin and mucosa, it is also a major pathogen causing harmful infections such as abscesses, pneumonia, and septicemia [1]. In fact, *S. aureus* is the leading cause for surgical site infections and especially methicillin-resistant *S. aureus* (MRSA) infections are associated with significant costs for health care systems [2].

Hyper-IgE syndromes (HIES) are a group of inborn errors of immunity (IEI), of which the most frequent form is caused by monogenic defects in the gene *STAT3* (STAT3-HIES) [3–5]. STAT3-HIES is characterized by elevated serum IgE levels, eosinophilia, eczema and increased susceptibility to *S. aureus* infections [4–6]. Severe staphylococcal skin and lung infections are common in STAT3-HIES patients, most likely because of the impairment in the epithelial immune response [7–9]. *S. aureus* can also cause infections in immunocompetent individuals; the predisposition of STAT3-HIES patients towards this pathogen, however, offers further insights into the immune defense against *S. aureus*. STAT3-HIES patients show impaired Th17 cell function and diminished memory B cell development [10–13]. Additionally, STAT3-HIES patients fail to raise antibodies against *S. aureus* toxin despite chronic colonization [14]. Importantly, besides an impaired humoral and cellular immune response towards *S. aureus*, STAT3-HIES patients also suffer from reduced epithelial immunity towards this pathogen [15]. STAT3-HIES patients benefit from Immunoglobulin replacement therapy (IgRT), most likely because it partly compensates the compromised humoral and cellular immune response [16, 17]. Nevertheless, new therapeutic strategies are needed to improve the clinical outcome of patients.

Although *S. aureus* colonization and infection are key symptoms of STAT3-HIES and patients frequently require antibiotic prophylaxis or therapy, little is known about the molecular features of *S. aureus* in these patients. Moreover, it is unclear if the immunodeficiency of STAT3-HIES patients selects *S. aureus* strains with a specific “genetic fingerprint”. A recent study used multi-locus sequence typing (MLST) and protein A (*spa*) typing to analyze the genetic background of 13 *S. aureus* isolates collected from STAT3-HIES patients in the USA [8]. Here, most isolates resembled highly virulent USA300 strains that are prevalent in hospitals in the USA and have been associated with MRSA outbreaks. The aim of our study was to analyze the *S. aureus* carriage profile of STAT3-HIES patients in Germany to optimize antibiotic treatment using bacterial whole genome sequencing (WGS) and to investigate virulence and resistance genes in these isolates.

## Methods

### Patients

All four STAT3-HIES patients carried a heterozygous dominant-negative *STAT3* mutation, were unrelated, and presented with the characteristic clinical findings of their genetically confirmed diagnosis at inclusion of study (Table 1). Mutations were reported using the nomenclature of den Dunnen and Antonarakis [19].

**Table 1:** Demographic and clinical information of STAT3-HIES patients

	STAT3-HIES patient 1 (P1)	STAT3-HIES patient 2 (P2)	STAT3-HIES patient 3 (P3)	STAT3-HIES patient 4 (P4)
Age	9 years of age	29 years of age	53 years of age	5 years of age
Mutation	c.1144C > T p.R382W	c.1145C > A p.R382Q	c.1144C > T p.R382W	c.1825A > G p.R609G
History of skin abscesses/eczema	+	+	+	+
History of recurrent respiratory infections	−	+	+	+
Antibiotic prophylaxis	Co-Trimoxazole Cephalexin	Co-Trimoxazole	−	Co-Trimoxazole

### *S. aureus* Isolate Collection, Whole Genome Sequencing, and Data Analysis

*S. aureus* strains were collected and cultured using standard procedures. In total eleven *S. aureus* isolates were collected including screening isolates (nose/throat *n* = 4; perianal *n* = 1) isolates collected from skin lesions (*n* = 5) and one clinical isolate from a lymph node abscess (please see Table 2 for further isolate and collection site details). Genomic DNA of *S. aureus* isolates was purified using the MagAttract HMW DNA kit (Qiagen, Venlo Netherlands) following manufacturer’s instructions. Isolates were sequenced using Illumina technology and Nextera XT version 2 chemistry, with a 250-bp paired-end protocol on a MiSeq sequencer (Illumina, San Diego, USA). Quality trimming of fastq files (average base quality of 30, aiming for 100-fold coverage) and de novo assembly using SKESA [20] were performed with SeqSphere + (version 6; Ridom GmbH, Münster, Germany) [21]. Only genomes harboring ≥ 95% core genome multi-locus sequence typing (cgMLST) targets of the *S. aureus* cgMLST scheme passed quality control; otherwise, sequencing was repeated. Target gene sets for virulence factors, resistance, and toxin genes as well as *spa* type were analyzed using the SeqSphere + software [21, 22]. Presence of enterotoxin genes was confirmed by polymerase chain reaction as described previously [23].

**Table 2:** Typing results of *S. aureus* isolates collected from STAT3-HIES patients

Patient	Sample	MLST ST	CC	*spa* type	*mecA*	*pvl*	Collection site	Collection date
P1	P1.1	5	5	t179	+	−	Nose/throat, screening swab	2011
	P1.2	7	-	t091	−	−	Lymph node abscess	2019
	P1.3	5	5	t179	−	−	Nose/throat, screening swab	2019
	P1.4	5	5	t179	−	−	Perianal screening swab	2019
P2	P2.1	97	97	t521	−	−	Nose/throat, screening swab	2012
P3	P3.1	582	15	t084	−	−	Skin lesion (left eyebrow)	2011
	P3.2	582	15	t084	−	−	Skin lesion (left arm)	2011
	P3.3	582	15	t084	−	−	Skin lesion (left ear)	2011
	P3.4	582	15	t084	−	−	Skin lesion (nose)	2011
	P3.5	582	15	t084	−	−	Skin lesion (right ear)	2011
P4	P4.1	45	45	t015	−	−	Nose/throat, screening swab	2019

## Results

### Patient Characteristics and *S. aureus *Isolates

Patients included in our study were between 5 and 53 years of age at enrollment. All four patients had a history of pulmonary and skin infections (Table 1). During the study period, patients received prophylactic antibiotic treatment as specified in Table 1 and skin treatment with octenidine dihydrochloride as well as symptomatic skin care with emollients. Eleven *S. aureus* isolates were collected either as screening isolates (nasal carriage) or from different skin sites. One clinical isolate (P1.2) was isolated from a lymph node abscess.

### Epidemiology of *S. aureus* Strains and *spa* Typing Results

All bacteria were analyzed for MLST, clonal complex (CC) and *spa* type. The collected *S. aureus* isolates were predominantly ST5 or ST582 and the most frequent spa types were t179 and t084 (Table 2). Only one isolate out of eleven was identified as MRSA. In addition, none of the isolates carried the Panton-Valentine leukocidin (*pvl)*, staphylococcal enterotoxins Q (*seq*), or staphylococcal enterotoxins K (*sek*) gene (Tables 2 and 3). These typing results match the general epidemiology of *S. aureus* in Germany and differ from previous reports that identified mostly MRSA isolates encoding the *pvl* gene in STAT3-HIES patients from the USA [24].

**Table 3:** Toxin and virulence genes in *S. aureus* isolates

	P1.1	P1.2	P1.3	P1.4	P2	P3.1	P3.2	P3.3	P3.4	P3.5	P4
	*agrIIB agrIIC agrIID*	*agrIB agrIC agrID*	*agrIIB agrIIC agrIID*	*agrIID*	*agrIB agrIC agrID*	*agrIIB agrIIC agrIID*	*agrIIB agrIID*	*agrIIB agrIIC agrIID*	*agrIIB agrIID*	*agrIIB agrIIC agrIID*	*agrIB agrID*
Hemolysin α (*hla*)	+	+	+	+	+	+	+	+	+	+	+
Hemolysin β (*hlb*)	−	−	−	−	−	−	−	−	−	−	−
Hemolysin δ (*hld*)	+	+	+	+	+	+	+	+	−	+	+
Hemolysin (*hl*)	+	+	−	−	+	+	+	+	+	+	+
γ hemolysin component A (*hlgA*)	+	+	+	+	+	+	+	+	+	+	+
γ hemolysin component B (*hlgB*)	+	+	+	+	−	+	+	+	+	+	+
γ hemolysin component C (*hlgC*)	+	+	+	+	+	+	+	+	+	+	+
Hemolysin (*hlIII*)	+	+	+	+	+	+	+	+	+	+	+
*saeS*	+	+	+	+	+	+	+	+	+	+	+
Transcriptional regulator (*sarA*)	+	+	+	+	+	+	+	+	+	+	+
*varS*	+	+	+	+	+	+	+	+	+	+	+
Enterotoxin A (*sea*)	+	−	+	+	−	−	−	−	−	−	−
Chemotaxis inhibitoy protein (*chp*)	+	−	+	+	+	−	+	+	−	+	+
Staphylococcal complement inhibitor (*scn*)	+	+	+	+	+	?	+	+	+	+	+
Staphylokinase (*sak*)	+	+	+	+	+	−	−	−	−	−	+
Serine protease A (*sspA*)	+	−	+	−	+	+	−	+	−	+	+
Serine protease B (*sspB*)	+	+	+	+	+	−	+	+	+	+	+
Enterotoxin b (*seb*)	−	−	−	−	−	−	−	−	−	−	−
Enterotoxin c (*sec*)	−	−	−	−	−	−	−	−	−	−	−
Enterotoxin d (*sed*)	−	−	−	−	−	−	−	−	−	−	−
Enterotoxin e (*see*)	−	−	−	−	−	−	−	−	−	−	−
Enterotoxin g (*seg*)	+	−	+	+	−	−	−	−	−	−	−
Enterotoxin h (*seh*)	−	−	−	−	−	−	−	−	−	−	−
Enterotoxin i (*sei*)	−	−	−	−	−	−	−	−	−	−	−
Enterotoxin j (*sej*)	−	−	−	−	−	−	−	−	−	−	−
Enterotoxin k (*sek*)	−	−	−	−	−	−	−	−	−	−	−
Enterotoxin l (*sel*)	−	−	−	−	−	−	−	−	−	−	−
Enterotoxin m (*sem*)	+	−	+	−	−	−	−	−	−	−	−
Enterotoxin n (*sen*)	+	−	+	−	−	−	−	−	−	−	−
Enterotoxin o (*seo*)	−	−	−	−	−	−	−	−	−	−	−
Enterotoxin q (*seq*)	−	−	-	−	−	−	−	−	−	−	−
Enterotoxin r (*ser*)	−	−	-	−	−	−	−	−	−	−	−
Enterotoxin u (*seu*)	−	−	+	−	−	−	−	−	−	−	−
Clumping factor B (*clfB*)	+	+	−	+	+	+	+	+	+	+	+
Elastin-binding protein (*ebpS*)	+	+	+	+	+	+	+	+	+	+	+
Fibronectin-binding protein (*fnbA*)	+	+	+	+	+	+	+	+	+	+	+
Intracellular adhesion protein A (*icaA*)	+	+	+	+	+	+	+	+	+	+	+
Intracellular adhesion protein C (*icaC*)	+	+	−	+	−	+	+	+	−	+	+
Intracellular adhesion protein D (*icaD*)	+	+	+	+	+	+	+	+	+	+	+
Exfoliative toxin A (*etA*)	−	−	−	−	+	+	−	−	−	−	−
Exfoliative toxin B (*etB*)	−	−	−	−	−	−	−	−	−	−	−
Exfoliative toxin D (*etD*)	−	−	−	−	−	−	−	−	−	−	−
Toxic shock syndrome toxine (*tst1)*	−	−	−	−	−	−	−	−	−	−	−
Leucotoxin D (*lukD*)	+	+	+	+	+	+	+	+	+	+	−
Leucotoxin E (*lukE*)	+	+	+	+	+	+	+	+	+	+	−

### Clonal Relationship of *S. aureus* Isolates in STAT3-HIES Patients

**Fig. 1:**
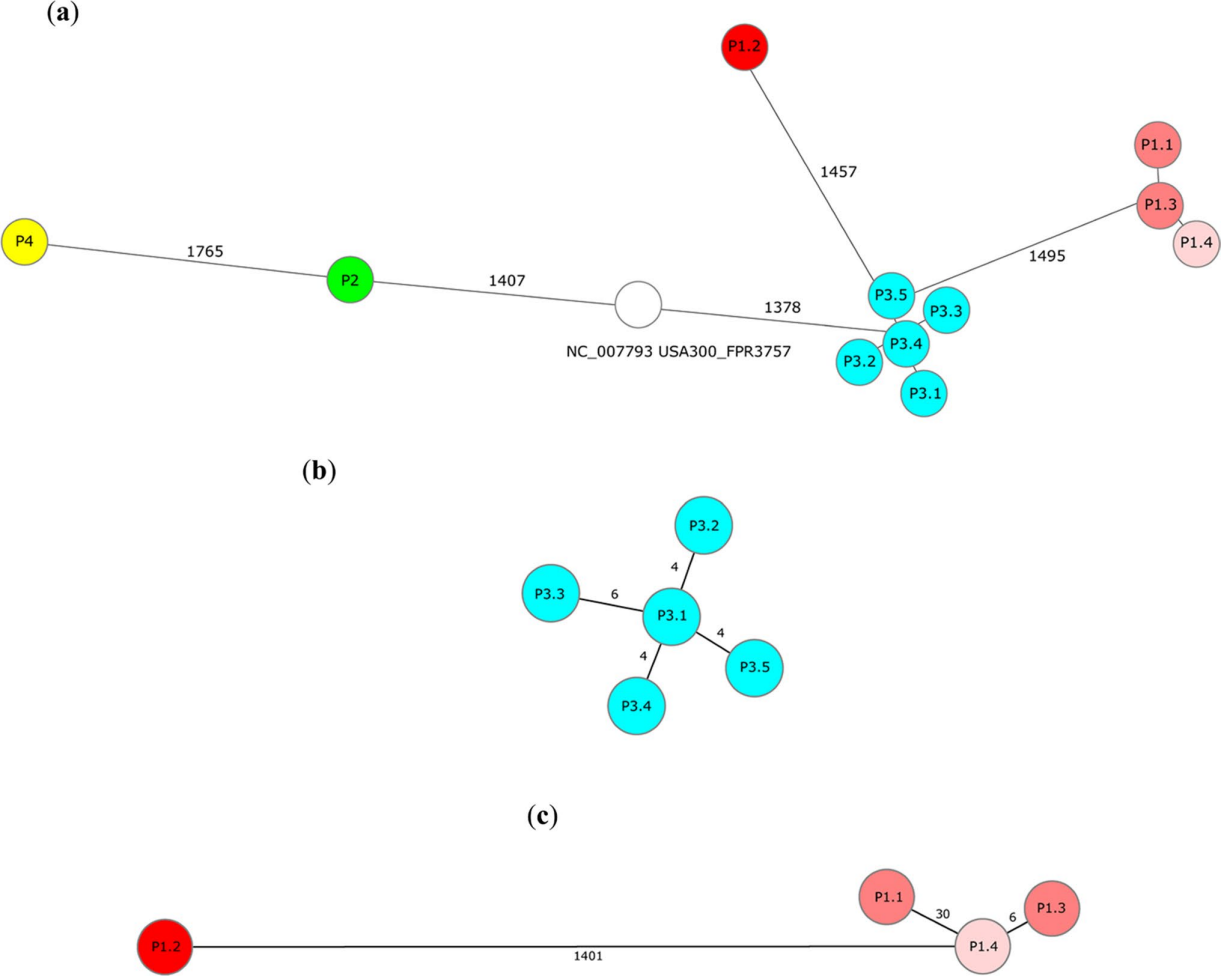
Minimum spanning trees of *S. aureus* isolates illustrate their genotypic relationship. Minimum spanning trees were based on up to 1861 cgMLST target genes, pairwise ignoring missing values. Every circle represents one genotype while connecting lines represent the number of different alleles in a pairwise comparison. **a** *S. aureus* isolates of different STAT3-HIES patients (P1–P4, using a different color for each patient). A reference sequence of a reference USA300 strain (NC_007793.1) was included as comparison (white). **b** The skin colonizing isolates of STAT3-HIES patient P3 (blue). **c** The isolate causing a lymph node abscess in STAT3-HIES patient P1 (P1.2, dark red) in comparison to three other isolates of the same patient. Isolates P1.1 and P1.3 are both isolated from a nose/throat screening swab (light red) but were collected 8 years apart. Isolate P1.4 (pink, perianal screening swab) has been collect at the same time point as P1.3

Next, the genetic relatedness of *S. aureus* isolates collected from STAT3-HIES patients was analyzed by cgMLST. cgMLST combines “classical” typing approaches to classify bacteria with the extensive genetic data sets obtained by WGS [25]. For *S. aureus* up to 1861 conserved target genes are compared to discriminate between isolates achieving a sufficient resolution for epidemiological and surveillance studies [26]. The isolates collected as part of this study were genetically unrelated and differed significantly from a reference sequence of highly virulent USA300 (NC_007793.1) (Fig. 1a). In one STAT3-HIES patient (P3) five *S. aureus* isolates were isolated from skin lesions at different sites yet at the same time point (P3.1 − P3.5). WGS showed that these isolates belonged to the same ST (ST582) and *spa* type (t084) but differed by up to 6 alleles or 6 single nucleotide polymorphisms (SNPs) in their core genome (Fig. 1b). In another STAT3-HIES patient (P1) WGS illustrated that the *S. aureus* isolate causing a lymph node infection (P1.2) was not genetically related to the clones colonizing the patient at other sites and differed from colonizing isolates in 1401 alleles or 9382 SNPs (Fig. 1c). The colonizing isolates P1.1, P1.3, and P1.4 belong to the same genetic family, ST (ST5) and *spa* type (t179), yet showed significant variation in their core genome. Strains P1.1 and P1.3 were both isolated from a nose/throat screening swab but were collected 8 years apart and differed by 30 alleles or 31 SNPs in their core genome. Taken together the analyses of these bacterial isolates indicate that STAT3-HIES patients are carrying several genetically diverse *S. aureus* isolates at a time.

### Pathogenicity Factors in Patients’ *S. aureus* Strains

**Fig. 2:**
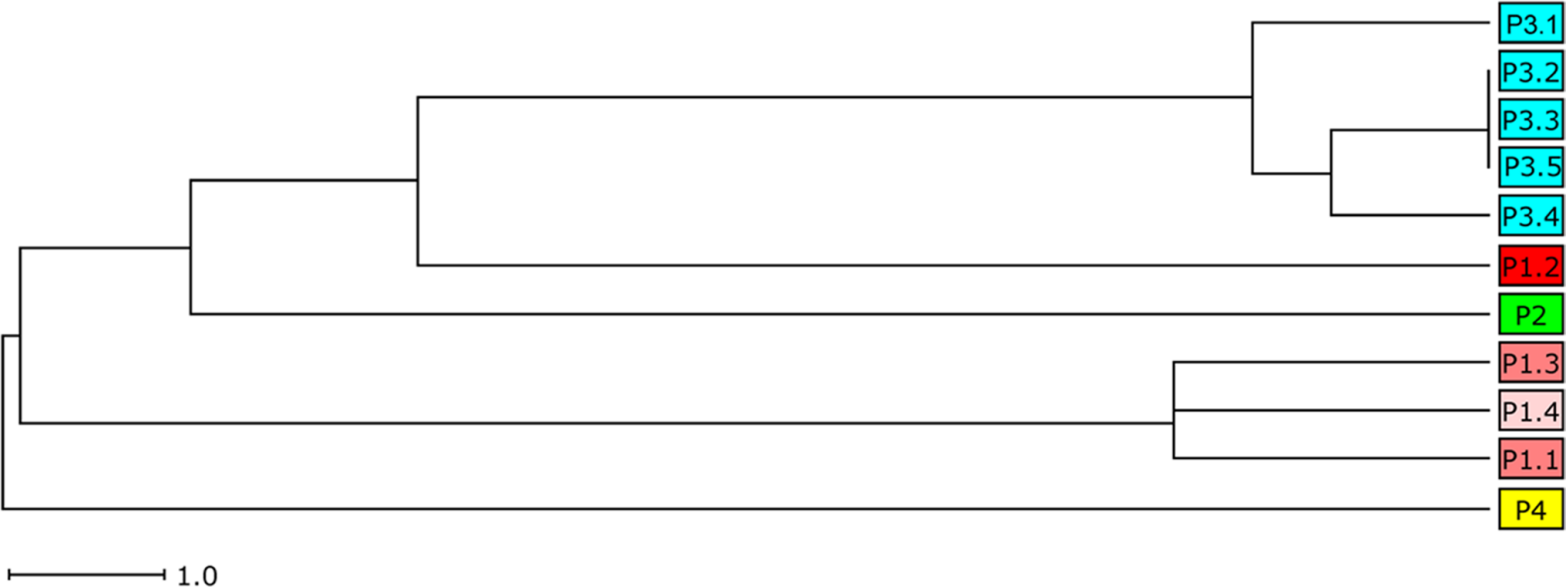
UPGMA-tree based on the toxin and virulence gene profile of eleven *S. aureus* isolates. The tree was drawn to scale with branches given in absolute alleles distance, using a different color for each patient (colors correspond to Fig. 1)

Several studies have correlated the ability of *S. aureus* to cause soft tissue infection and inflammation with the activity of pathogenicity factors and bacterial toxin genes [22]. Therefore, we analyzed the *S. aureus* isolates of STAT3-HIES patients for genes of the accessory regulator (*agr*), hemolysin genes (*hl*), and genes encoding for enterotoxins, adhesion molecules and the immune evasion complex (IEC) (Table 3). In addition, we constructed a unweighted pair group method with arithmetic mean (UPGMA)-tree based on their toxin and virulence gene profile of the *S. aureus* isolates to illustrate the distribution pattern of the 45 pathogenicity factors analyzed in the study (Fig. 2).

Hemolysin α (*hla*) was present in all isolates. Two colonizing isolates showed a stop codon in the *hl* gene (P1.3 and P1.4). One colonizing isolate lacked the hemolysin δ (*hld*) gene (P3.4). The IEC consists of the genes coding for staphylokinase (*sak*), staphylococcal complement inhibitor (*scn*), chemotaxis inhibitory protein (*chp*), and staphylococcal enterotoxins A *(sea*). IEC genes are well-known pathogenicity factors that aid *S. aureus* to bypass the human immune response by counteracting key steps in innate immunity such as complement activation and chemotaxis. Interestingly, three of the eleven isolates collected from STAT3-HIES patients contained all four IEC genes (isolates P1.1, P1.3 and P1.4, all isolated from patient P1). As STAT3-HIES patients show reduced Th17 and B cell responses towards *S. aureus*, bacterial pathogenicity factors that impair innate immunity might result in a more pronounced effect in these patients. However, long-term follow-up studies are needed to address this research question.

## Discussion

Presently, the molecular characteristics and genetic variability of *S. aureus* clones in STAT3-HIES patients remain largely unknown. The MLST ST and *spa* types of the eleven *S. aureus* isolates collected during this study differed significantly from the typing results described in a previous report from the USA [18]. However, the identified ST and *spa* types are common in Germany [22). The ST and *spa* type results imply that the local epidemiology is the main factor determining which *S. aureus* strains colonize STAT3-HIES patients. Although all patients enrolled in our study have received long-time antibiotic prophylaxis and therapy, MRSA was not prevalent in our patient cohort (Table 2). In fact, MRSA infections in the healthcare setting have been decreasing steadily over the recent years in Germany [27, 28]. In 2018, a surveillance conducted by Germany’s public health institution, the Robert Koch-Institute, estimated that 7.7% of all *S. aureus* bacteria isolated from patients in the community setting are MRSA [29]. Further studies are required to investigate if the prevalence of MRSA among STAT3-HIES patients equals the prevalence of the general population in their home countries. Yet at present, we recommend that empirical antibiotic treatment for severe infection in STAT3-HIES patients might not have to cover MRSA unless a patient is a known carrier or the local MRSA prevalence is high. Despite the limitation of relative low patient number of patients enrolled, due to STAT3-HIES being a rare disease, our study shows first interesting results how WGS can be used to characterize *S. aureus* isolates collected from STAT3-HIES patients. In our study, we analyzed the genetic relatedness of colonizing *S. aureus* isolates from several STAT3-HIES patients based on WGS data. Here, we demonstrated that STAT3-HIES patients were colonized with genetically diverse *S. aureus* clones at a time. Similar observations have been made in a study analyzing *S. aureus* isolates collected from eczematic lesions of atopic eczema patients by WGS. In this publication, a broad genetic diversity of bacterial isolates was detected suggesting that clonal expansion of a bacterial population takes place during a disease flare [30].

The WGS data of *S. aureus* isolates generated as part of this study offer a comprehensive characterization of multiple virulence factors. An improved understanding of pathogenicity factors may pave the way for new therapeutic strategies for STAT3-HIES patients. Currently, long-acting monoclonal antibodies that are capable to neutralize *S. aureus* toxins in the respiratory tract are under development to treat MRSA lung infections [31, 32]. As STAT3-HIES patients show reduced IgG levels for *S. aureus* toxins, the potential of these monoclonal antibodies as treatment for severe *S. aureus* lung infections could be evaluated [14, 33].

Taken together, our study provides a detailed view into the molecular characteristics of the *S. aureus* isolates collected from STAT3-HIES patients, including genetic relatedness of isolates, methicillin resistance and presence of virulence factors and demonstrates the benefits of using molecular approaches to study host–pathogen dynamics in IEI.

## Data Availability

The whole genome datasets of *S. aureus* isolates generated for this study have been deposited in the European Nucleotide Archive (ENA) at EMBL-EBI under accession number PRJEB52061 (https://www.ebi.ac.uk/ena/browser/view/PRJEB52061).

## Abbreviations

*agr*: Accessory gen regulator
cgMLST: Core genome multi-locus sequence typing
*chp*: Chemotaxis inhibitory protein of staphylococcus
CC: Clonal complex
HIES: Hyper-IgE syndromes
*hl*: Hemolysin
*hla*: Hemolysin α
*hld*: Hemolysin δ
IEC: Immune evasion cluster
IgRT: Immunoglobulin replacement therapy
IEI: Inborn error of immunity
MRSA: Methicillin-resistant *S. aureus*
MLST: Multi-locus sequence typing
*pvl*: Panton-Valentine leukocidin
*spa*: Protein A
*scn*: Staphylococcal complement inhibitor
*sea*: Staphylococcal enterotoxins A
*sek*: Staphylococcal enterotoxins K
*seq*: Staphylococcal enterotoxins Q
*S. aureus*: *Staphylococcus aureus*
*sak*: Staphylokinase
UPGMA: Unweighted pair group method with arithmetic mean
WGS: Whole genome sequencing

## Code Availability

The code related to data analysis is available from the corresponding author on request.
